# Dorsal root ganglion magnetic resonance imaging biomarker correlations with pain in Fabry disease

**DOI:** 10.1093/braincomms/fcae155

**Published:** 2024-05-01

**Authors:** Magnus Schindehütte, Simon Weiner, Katharina Klug, Lea Hölzli, Christopher Nauroth-Kreß, Florian Hessenauer, Thomas Kampf, György A Homola, Peter Nordbeck, Christoph Wanner, Claudia Sommer, Nurcan Üçeyler, Mirko Pham

**Affiliations:** Department of Neuroradiology, University Hospital Würzburg, Würzburg 97080, Germany; Department of Neuroradiology, University Hospital Würzburg, Würzburg 97080, Germany; Department of Neurology, University Hospital Würzburg, Würzburg 97080, Germany; Department of Neuroradiology, University Hospital Würzburg, Würzburg 97080, Germany; Department of Neuroradiology, University Hospital Würzburg, Würzburg 97080, Germany; Department of Neuroradiology, University Hospital Würzburg, Würzburg 97080, Germany; Department of Neuroradiology, University Hospital Würzburg, Würzburg 97080, Germany; Department of Neuroradiology, University Hospital Würzburg, Würzburg 97080, Germany; Department of Internal Medicine, University Hospital Würzburg, Würzburg 97080, Germany; Department of Internal Medicine, University Hospital Würzburg, Würzburg 97080, Germany; Department of Neurology, University Hospital Würzburg, Würzburg 97080, Germany; Department of Neurology, University Hospital Würzburg, Würzburg 97080, Germany; Department of Neuroradiology, University Hospital Würzburg, Würzburg 97080, Germany

**Keywords:** magnetic resonance gangliography, magnetic resonance neurography, magnetic resonance imaging, neuropathic pain, peripheral neuropathy

## Abstract

Fabry disease is a rare monogenetic, X-linked lysosomal storage disorder with neuropathic pain as one characteristic symptom. Impairment of the enzyme alpha-galactosidase A leads to an accumulation of globotriaosylceramide in the dorsal root ganglia. Here, we investigate novel dorsal root ganglia MR imaging biomarkers and their association with Fabry genotype and pain phenotype. In this prospective study, 89 Fabry patients were examined using a standardized 3 T MRI protocol of the dorsal root ganglia. Fabry pain was assessed through a validated Fabry pain questionnaire. The genotype was determined by diagnostic sequencing of the alpha-galactosidase A gene. MR imaging end-points were dorsal root ganglia volume by voxel-wise morphometric analysis and dorsal root ganglia T2 signal. Reference groups included 55 healthy subjects and Fabry patients of different genotype categories without Fabry pain. In patients with Fabry pain, T2 signal of the dorsal root ganglia was increased by +39.2% compared to healthy controls (*P* = 0.001) and by +29.4% compared to painless Fabry disease (*P* = 0.017). This effect was pronounced in hemizygous males (+40.7% compared to healthy; *P* = 0.008 and +29.1% compared to painless; *P* = 0.032) and was consistently observed across the genotype spectrum of nonsense (+38.1% compared to healthy, *P* < 0.001) and missense mutations (+39.2% compared to healthy; *P* = 0.009). T2 signal of dorsal root ganglia and globotriaosylsphingosine levels were the only independent predictors of Fabry pain (*P* = 0.047; *P* = 0.002). Volume of dorsal root ganglia was enlarged by +46.0% in Fabry males in the nonsense compared to missense genotype category (*P* = 0.005) and by +34.5% compared to healthy controls (*P* = 0.034). In painful Fabry disease, MRI T2 signal of dorsal root ganglia is increased across different genotypes. Dorsal root ganglion MRI T2 signal as a novel *in vivo* imaging biomarker may help to better understand whether Fabry pain is modulated or even caused by dorsal root ganglion pathology.

## Teaser

Schindehütte *et al.* report that in Fabry disease, MRI of dorsal root ganglia reveals significant correlations with Fabry pain phenotype. Increased dorsal root ganglion T2 signal intensity and volume changes were associated with the Fabry pain phenotype and genotypes of varying severity, providing insight into the pathophysiology of Fabry-related pain.

## Graphical Abstract

**Graphical Abstract:**
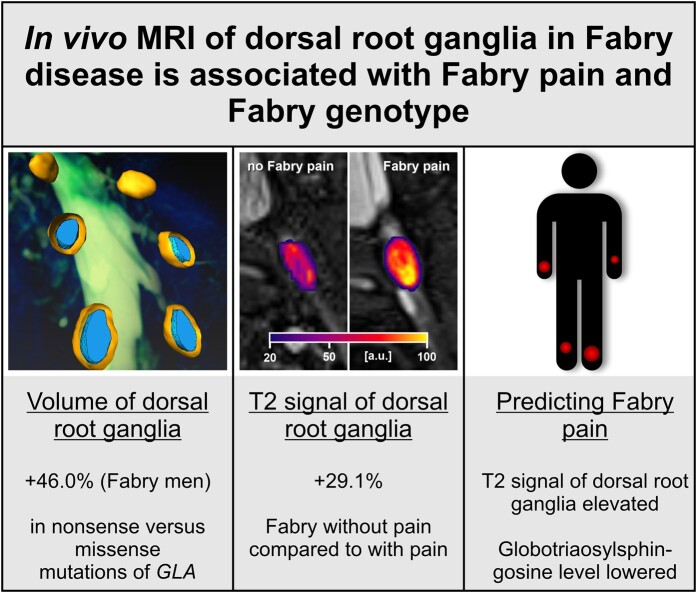

## Introduction

Fabry disease is an X-linked lysosomal storage disorder, caused by mutations in the alpha-galactosidase A (*GLA*) gene encoding the GLA enzyme.^1^ Consecutive loss or reduction of GLA activity leads to an accumulation of glycosphingolipids, primarily globotriaosycleramide (Gb3) and its deacylated derivative globotriaosylsphingosine (Lyso-Gb3) in several cell types, including neurons and endothelial cells.^2^

Episodic and acral pain is one of the most debilitating and early symptoms in Fabry disease already starting during childhood. The pathophysiology of pain in Fabry disease is incompletely understood, and Fabry pain is often not responsive to Fabry disease treatment.^3^ Although small fibre pathology with peripheral denervation and loss of cold sensation is a hallmark of Fabry disease, objective *in vivo* biomarkers that are associated with Fabry pain are missing. Such biomarkers are highly desirable not only for offering insights into the pathophysiology of Fabry pain but potentially also for the development and clinical testing of novel therapeutics such as second generation enzyme replacement therapy, substrate reduction therapy as well as mRNA- and gene-based therapy.^4^

Our efforts in finding novel imaging biomarkers of Fabry pain are guided by several experimental observations suggesting that the dorsal root ganglion (DRG) may play a key role in the development and persistence of pain in Fabry disease.^3^ Specifically, in the *Gla* knockout mouse model of Fabry disease, Gb3 deposits were associated with a loss of sodium currents in DRG sensory neurons, which was reversible upon Gb3 cleavage.^5^ Furthermore, in the *Gla* knockout mouse model, an interplay between Gb3 accumulation, altered ion channel expression and pain-like behaviour was reported.^6^ However, so far, severe methodical limitations impede to observe the DRG by *in vivo* imaging in the human system or in animal experimental settings.

Recently, high-resolution DRG MRI has been established as a new imaging technique to observe the DRG *in vivo.*^7-11^ DRG MRI allows morphometric, microstructural and functional analysis of the DRG, e.g. by the technique of microvascular MRI perfusion. DRG MRI provided first evidence of enlarged DRG volume in Fabry disease patients in early studies.^10^ However, these early observations in a smaller cohort did not include patients with and without Fabry pain and did not investigate changes in the DRG between the different Fabry disease patient groups with different underlying mutations.

We now investigated voxel-wise DRG MRI morphometry and DRG T2 signal as potential *in vivo* imaging biomarkers. We observed these end-points in the largest Fabry disease cohort so far studied with DRG imaging. We also performed subanalyses based on different Fabry disease genotypes. Our hypotheses are that DRG volume and DRG T2 signal may serve as *in vivo* imaging biomarkers indicating DRG pathology in Fabry disease and that DRG imaging pathology is associated with Fabry pain.

## Materials and methods

### Clinical data collection

For this prospective observational study, 89 Fabry disease patients were recruited between 01/2017 and 08/2021. Fifty-five healthy volunteers were prospectively recruited for this study and served as a control group. Inclusion criteria for healthy controls were no neurological disease, absence of neuropathic pain or other sources of pain as well as absence of any contraindications for MRI.

Fabry disease patients were recruited through one of the largest Fabry disease centres, the Fabry Centre for interdisciplinary Treatment (FAZiT) at the University Hospital Würzburg. Complete Fabry disease genotype and phenotype classification could be accomplished in all Fabry disease participants.^12,13^

All healthy controls underwent the same MRI examination protocol as Fabry disease patients.

### Classification of Fabry disease genotype and Fabry pain phenotype

Complete determination of specific genotype variants resulted in the following classification: (i) missense-; (ii) nonsense-; (iii) intron-; or (iv) otherwise-classifiable Fabry disease-related *GLA* mutations. The evaluation of the Fabry pain phenotype was accomplished through an established pain questionnaire that was designed and validated for Fabry pain in adults, the Würzburg Fabry Pain Questionnaire (FPQ).^14^ Patients were assigned to the ‘Fabry pain’ (FDp) group according to their affirmation of a combination of Fabry disease-specific items on the FPQ: presence of pain attacks or chronic pain with associated pathognomonic acral burning pain at present or in the past. All other Fabry disease patients without pathognomonic Fabry pain history were assigned to the ‘no Fabry pain’ (FDn) group.

### Assignment of genotype to clinical phenotype

Clinical Fabry disease disease phenotype classification comprehensively included all organ systems and served to associate the overall clinical phenotype with the genotype category. Overall clinical Fabry disease phenotype, according to the currently established classification, includes ‘classic/early-onset Fabry disease’, ‘non-classic/late-onset Fabry disease’ and ‘benign Fabry disease’.^12^ Typical classic/early-onset Fabry disease is defined by complete absence of residual GLA activity and this most severe form is, in addition to Fabry pain, also strongly associated with hypertrophic cardiomyopathy, renal failure and cerebral stroke. Non-classic Fabry disease, also referred to as later-onset Fabry disease, is defined by isolated symptoms, typically confined to a single organ, and also by delayed clinical manifestation not until adulthood.

The third category of ‘benign Fabry disease’ is defined by preserved or only slightly impaired enzyme function without clinically evident organ manifestations.^15^

Eventually, objective assignment between Fabry disease genotype and definite phenotype was achieved through using the public Fabry database. Its information represents *a priori* knowledge by which exact links between the molecular structural enzyme alteration to genotype *GLA* mutation variant and clinical phenotype can be established. In all but three participants, Fabry disease genotype could be exactly linked to clinical phenotype category (fabry-database.org).^16^ For these three remaining patients, neither literature nor the Fabry database provided a confirmed unambiguous assignment of genotype to phenotype, and therefore these three cases were exempt from phenotype classification. A detailed overview of the Fabry disease genotype phenotype classification is shown in Table 1. An overview of all Fabry disease patients including detailed information about genotype, clinical phenotype and Fabry pain status is given in Supplementary Table 1.

**Table 1:** Distribution of subgroups within Fabry disease patients

	Fabry pain phenotype [*n* (%)]	No Fabry pain phenotype [*n* (%)]
All Fabry disease patients	45 (50.6)	44 (49.4)
Sex		
Female	23 (51.1)	27 (61.4)
Male	22 (48.9)	17 (38.6*)*
Fabry disease genotype		
Nonsense mutation	14 (31.1)	5 (11.4)
Missense mutation	25 (55.6)	37 (84.1)
Intron mutation	4 (8.9)	1 (2.3)
Otherwise classifiable	2 (4.4)	1 (2.3)
Clinical phenotype		
Classic	31 (68.9)	7 (15.9)
Later-onset	5 (11.1)	20 (45.5)
Benign	2 (4.4)	14 (31.8)
Unambiguous	7 (15.6)	3 (6.8)

Descriptive analysis of all Fabry disease patients (*n* = 89) according to their Fabry pain phenotypes with respect to sex, Fabry disease genotype and clinical phenotype based on literature and Fabry database (fabry-database.org). DRG, dorsal root ganglion.

### Imaging protocol

All DRG MRI examinations were conducted on 3 tesla MRI scanners between January 2017 and August 2021 at the Department of Neuroradiology, Würzburg University Hospital, Germany (Magnetom PRISMAfit or Magnetom SKYRA, Siemens Healthineers, Erlangen, Germany). The DRG MRI protocol included the following pulse sequence: high-resolution 3D T2-w fast-spin-echo (FSE) sequence (SPACE: sampling perfection with application optimized contrasts using different flip angle evolution) with spectral fat saturation of the lumbosacral plexus and spine (scanning parameters: FOV 300 × 295 × 106 mm³, voxel size 1.0 × 1.0 × 1.0 mm³, ΔTE: 4.4 ms, TE: 301 ms, TR: 2000 ms). The imaging slab was aligned perpendicular to the L5 vertebral body and included bilaterally DRG levels L5 and S1 completely.

### Image analysis

DRG were analysed using the publicly available software packages for MR image analyses FSLeyes (McCarthy, Paul. FSLeyes) and napari (napari contributors (2019). napari: a multi-dimensional image viewer for Python. doi:10.5281/zenodo.3555620).

DRG volume estimation was performed by exact contour segmentation and automatic volume calculation of paired L5 and S1 DRG volumes. Exact contour segmentation was performed by two independent expert raters who were blinded to subject identity and group status using voxel-based volumetric analysis (total of 576 DRGs; L5: 288, S1: 288 in *n* = 144 subjects).^11^

To compensate for intra- and interindividual differences in the morphometry of DRG between different spinal levels, the sum of these four segmented lumbosacral DRG, which represent the two spinal levels with largest DRG volume in humans, was determined to obtain a single, representative DRG volume per subject [DRG volume (mm^3^)]. To determine the DRG T2 signal, these precise DRG regions of interest (ROIs) of the same four intraindividual L5 and S1 DRGs were analysed and a single mean average value was calculated and normalized to adjacent local cerebrospinal fluid (CSF). This local CSF average signal was then used for DRG T2 signal normalization by reading it out from a CSF-ROI measuring 160 voxels within the lumbosacral thecal sac. Signal homogeneity, molecular similarity to water and within-body anatomical proximity in relation to the target DRG are the major advantages in favour of using local CSF for T2 DRG signal normalization. DRG T2 signal normalization was not possible in only very few cases [*n* = 7 (*n* = 4 Fabry disease patients; *n* = 3 healthy controls)] either due to the anatomical constitution of the thecal sac or due to flow related artefacts within CSF. These subjects were excluded from DRG T2 signal analysis.

### Statistical analysis

Statistical analyses and data visualization were performed using GraphPad Prism (GraphPad Prism version 9.4.0, GraphPad Software, San Diego, CA, USA) and R version 4.3.2 (R Core Team. R: a language and environment for statistical computing. R Foundation for Statistical Computing, Vienna, Austria. URL https://www.R-project.org). Mean values for total DRG volume and mean DRG T2 signal were calculated for each subject. Testing for normal distribution was performed by qq plot and Kolmogorov–Smirnov test. Testing for differences between groups was performed using the chi-square test with Yates’ continuity correction for categorical variables or using the Wilcoxon–Mann–Whitney test, the unpaired *t*-test or, if appropriate, the one-way analysis of variance (ANOVA) with Tukey correction for continuous variables, respectively. Where appropriate, the correlation between parameters was calculated by Spearman’s correlation coefficient *r*. A multiple logistic regression analysis was performed to investigate the relationship between pain occurrence in Fabry disease and various predictors including the type of genetic mutation, body mass index (BMI), sex, DRG volume, DRG T2 signal and Lyso-Gb3 levels. The regression model was fitted using data transformed to optimize the model’s performance, with square root transformations for BMI, DRG volume and DRG T2 signal and logarithmic transformation for Lyso-Gb3 levels. The goodness of fit of the model was assessed using different types of pseudo-R-squared statistics [McFadden’s (McF *R*²), Maximum Likelihood (ML *R*²), Cragg and Uhler’s (CU *R*²)] and the Akaike information criterion (AIC). To validate the model, a bootstrap approach with 10 000 iterations was applied.^17^ To visualize the performance of the multiple logistic regression model, a receiver operating characteristic (ROC) curve was computed. Additionally, the area under the curve (AUC) was calculated to provide a single measure of the model’s overall accuracy. Probability values of *α* < 0.05 were considered statistically significant. Numerical group level data are expressed as mean values ± standard error of the mean with 95% confidence intervals where appropriate.

### Standard protocol approvals and patient consents

All data in this prospective study were collected in accordance with the Declaration of Helsinki and in compliance with the local ethics board. Written informed consent was obtained from all participants in the study. Approval was received from the Ethics Committee of the University of Würzburg to conduct the current study (IRB# 26/19-sc).

## Results

### Demographic patient data

Eighty-nine Fabry disease patients [50 females (56.2%), age: 46.6 ± 1.5 years] and 55 healthy subjects [34 females (61.8%), age: 37.8 ± 1.6 years] were included. Fabry disease patients and healthy controls were not 1:1 matched. Regarding sex (*X*^2^ = 0.243, *P* = n.s.), height (*t* = 0.687, *P* = n.s.), weight (*W* = 2377.5, *P* = n.s.) and BMI (*W* = 2296, *P* = n.s.), there was no significant difference between the two groups. However, there was a significant difference in age (*t* = −3.976, *P*  *<* 0.001). Detailed anthropometric data for Fabry disease patients are added as supplementary information (Supplementary Table 1).

### DRG T2 signal is elevated in Fabry disease patients with pain

**Figure 1:**
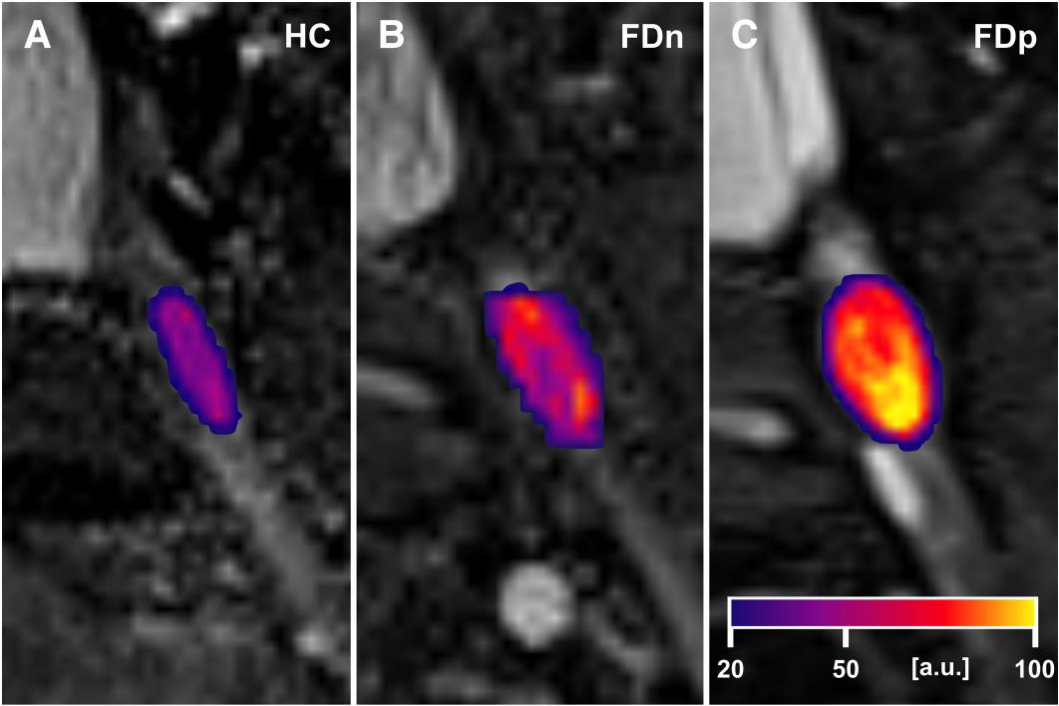
**Visualization of DRG T2 signal effect in Fabry pain**. Visualization of the DRG T2 signal effect in a patient with Fabry pain (**C**; T2 = 60.2 a.u.) compared to an Fabry disease patient without Fabry pain (**B**; T2 = 37.8 a.u.) and a healthy control (**A**; T2 = 33.5 a.u.). The heat maps represent the voxel-wise mapping of the normalized T2 signal of the region of interest (DRG level S1) in these representative subjects. Normalized DRG T2 signal intensity in arbitrary units (a.u.). HC, healthy control; DRG, dorsal root ganglion; FDn, no Fabry disease pain; FDp, Fabry disease pain; S1, sacral level 1.

One-way ANOVA showed a difference in DRG T2 signal between Fabry disease patients with Fabry pain, without Fabry pain and healthy controls (*F* = 6.9; *P* = 0.001). *Post hoc* comparison showed an increased DRG T2 signal in Fabry disease patients with Fabry pain compared to healthy controls (+39.2%; 49.7 versus 35.7 a.u.; *P* = 0.001). It was also higher in patients with Fabry pain compared to Fabry disease patients without pain (+29.4%; 49.7 versus 38.4 a.u.; *P* = 0.017). There was no difference between Fabry disease males and Fabry disease females with Fabry pain (47.0 versus 52.9 a.u.; *P* = n.s.; *t* = 0.78). Figure 1 visualizes this DRG T2 signal effect on the individual level of a representative single subject with Fabry pain (right image) versus a Fabry disease patient without Fabry pain (centre image) and versus a healthy control (left image).

One-way ANOVA showed a difference between Fabry disease males with Fabry pain, Fabry disease males without Fabry pain and healthy males (*F* = 5.7; *P* = 0.005). *Post hoc* comparison showed an increased DRG T2 signal in Fabry disease males with Fabry pain compared to Fabry disease males without Fabry pain (+40.7%; 47.0 versus 33.4 a.u.; *P* = 0.008). It was also higher in Fabry disease males with pain compared to healthy males (+29.1%; 47.0 versus 36.4 a.u.; *P* = 0.032). One-way ANOVA showed a difference between Fabry disease females with Fabry pain, Fabry disease females without Fabry pain and healthy females (*F* = 3.8; *P* = 0.027). *Post hoc* comparison showed a trend towards higher DRG T2 signal in Fabry disease females with Fabry pain compared to Fabry disease females without Fabry pain (+27.2%; 52.9 versus 41.6 a.u.; *P* = n.s.). It was higher in Fabry disease females with pain compared to healthy females (+49.9%; 52.9 versus 35.3 a.u.; *P* = 0.020).

Group comparisons testing a potential impact of genotype on the expression of imaging biomarkers for Fabry pain revealed the following.

One-way ANOVA showed a difference between Fabry disease patients carrying a nonsense mutation with Fabry pain, without pain and healthy controls (*F* = 5.6; *P* = 0.006). *Post hoc* comparison showed no difference in DRG T2 signal in Fabry disease patients carrying a nonsense mutation with Fabry pain compared to Fabry disease patients carrying a nonsense mutation without Fabry pain (+26.4%; 49.3 versus 39.0 a.u.; *P* = n.s.). It was higher in Fabry disease patients carrying a nonsense mutation with Fabry pain compared to healthy controls (+38.1%; 49.3 versus 35.7 a.u.; *P* = 0.004).

One-way ANOVA showed a difference between Fabry disease patients carrying a missense mutation with Fabry pain, without pain and healthy controls (*F* = 4.9; *P* = 0.010). *Post hoc* comparison showed an increased DRG T2 signal in Fabry disease patients carrying a missense mutation with Fabry pain compared to Fabry disease patients carrying a missense mutation without Fabry pain (+32.9%; 49.7 versus 37.4 a.u.; *P* = 0.036). It was higher in Fabry disease patients carrying a missense mutation with Fabry pain compared to healthy controls (+39.2%; 49.7 versus 35.7 a.u.; *P* = 0.009).

**Figure 2:**
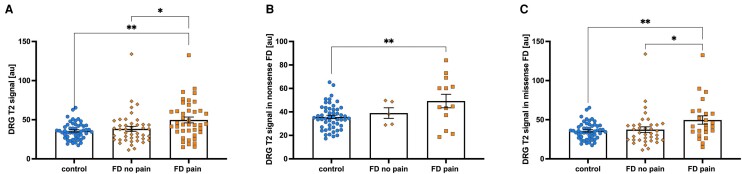
**DRG T2 signal in patients with and without Fabry pain**. (**A**) DRG T2 signal is elevated in patients with Fabry pain compared to patients without Fabry pain and healthy controls (49.7 versus 38.4 versus 35.7 a.u.). One-way ANOVA: *F* = 6.9; *P* = 0.001; (**B**) DRG T2 signal is increased in Fabry disease patients with pain and nonsense mutations of *GLA* (49.3 versus 39.0 versus 35.7 a.u.). One-way ANOVA: *F* = 5.6; *P* = 0.006; (**C**) DRG T2 signal is increased in Fabry disease patients with pain and missense mutations of *GLA* (49.7 versus 37.4 versus 35.7 a.u.). One-way ANOVA: *F* = 4.9; *P* = 0.010. DRG, dorsal root ganglion; FD, Fabry disease; a.u., arbitrary units.

Correlation analysis showed a correlation between the clinical phenotypes and the DRG T2 signal in Fabry disease patients (*r* = 0.36, *P* = 0.002). Figure 2 represents graphical representation of overall and within genotype group DRG T2 signal for each Fabry pain phenotype group and controls.

### DRG T2 signal and Lyso-Gb3 as independent predictors of Fabry pain

A multiple logistic regression analysis was performed to investigate the relationship between Fabry pain and various predictors, including Fabry disease genotype, BMI, sex, DRG volume, DRG T2 signal intensity and Lyso-Gb3 in Fabry disease patients. The regression model was fitted using data transformed to optimize model performance, with transformations such as square root for BMI, DRG volume, DRG T2 signal intensity and logarithmic transformation for Lyso-Gb3.

**Figure 3:**
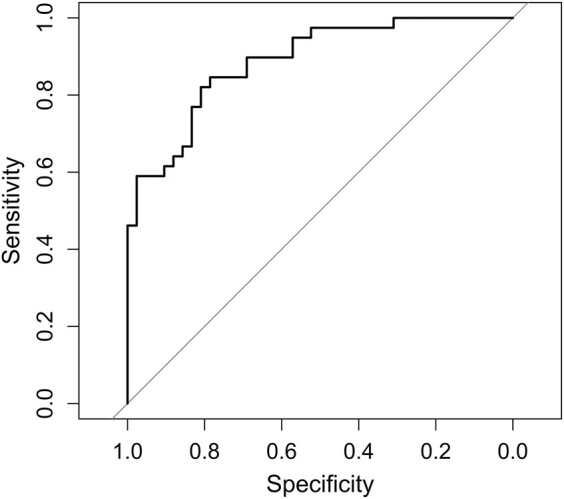
**ROC analysis for predicting Fabry pain phenotype in Fabry disease patients**. Analysis of the relationship between Fabry pain and possible predictors, including Fabry disease genotype, BMI, sex, DRG volume, DRG T2 signal intensity and Lyso-Gb3 levels. The regression model was fitted using data transformed to optimize model performance, with transformations such as square root for BMI, DRG volume, DRG T2 signal intensity and logarithmic transformation for Lyso-Gb3 levels. Coefficients of the model were estimated and showed that DRG T2 signal intensity (*P* = 0.047) and Lyso-Gb3 levels (*P* = 0.002) were the only significant predictors contributing to the model of Fabry pain. The ROC analysis underlines the performance of the model (AUC = 0.89). The goodness of fit of the model was assessed using the Akaike information criterion (AIC = 81.607) and pseudo-R-squared values (McF *R*^2^ = 0.397, ML *R*^2^ = 0.423, CU *R*^2^ = 0.565). The robustness of the model was confirmed by nonlinear bootstrapping (LLV = −33.80; B = 3.41; SE = 5.20). AIC, Akaike information criterion; McF *R*², McFadden’s pseudo-R-squared; ML *R*², Maximum Likelihood pseudo-R-squared; CU *R*², Cragg and Uhler’s pseudo-R-squared; BMI, body mass index; DRG, dorsal root ganglion; Lyso-Gb3, globotriaosylsphingosine; ROC, receiver operating characteristic.

A multiple logistic regression analysis identified two independent predictors for the occurrence of Fabry pain in Fabry disease patients: DRG T2 signal intensity (*P* = 0.047) and Lyso-Gb3 level (*P* = 0.002). Table 2 gives an overview of the predictive value of the individual independent variables. Assessed Akaike information criterion (AIC = 81.607) and pseudo-R-squared values (McF *R*^2^ = 0.397, ML *R*^2^ = 0.423, CU *R*^2^ = 0.565) indicated that the model explained a reasonable amount of variance in the occurrence of Fabry pain. The validation of the model revealed an original log-likelihood value of −33.80, with a bias of 3.41 and a standard error of 5.20 in the bootstrapped samples. A ROC curve with an AUC of 0.89 visualizes the performance of the multiple logistic regression model (Fig. 3).

**Table 2:** Coefficients of the multiple logistic regression model for predicting Fabry pain

Variable	Estimate	Std. error	Odds ratio	*P*-value
Intercept	−9.350	4.860	<0.001	0.054
Fabry disease genotype	−0.355	0.512	0.701	0.488
BMI	0.995	0.758	2.705	0.189
Sex	0.070	0.720	1.073	0.922
DRG volume	0.009	0.047	1.009	0.841
DRG T2 signal	0.397	0.200	1.487	0.047
Lyso-Gb3	1.175	0.375	3.238	0.002

Estimates, standard errors, odds ratios and *P*-values for each independent variable in the model were calculated (*n* = 89 Fabry disease patients). The dependent variable is the Fabry pain phenotype. Independent variables include Fabry disease genotype, BMI, sex, DRG volume, DRG T2 and Lyso-Gb3. A positive (negative) coefficient indicates an increase (decrease) in the log-odds of the dependent variable for each unit increase in the independent variable. Only DRG T2 and Lyso-Gb3 contributed to the model. The goodness of fit of the model was assessed using the Akaike information criterion (AIC = 81.607) and pseudo-R-squared values (McF *R*^2^ = 0.397, ML *R*^2^ = 0.423, CU *R*^2^ = 0.565). AIC, Akaike information criterion; BMI, body mass index; CU *R*², Cragg and Uhler’s pseudo-R-squared; DRG, dorsal root ganglion; Lyso-Gb3, globotriaosylsphingosine; McF *R*², McFadden’s pseudo-R-squared; ML *R*², Maximum Likelihood pseudo-R-squared.

Between the two identified independent predictors for the occurrence of Fabry pain in Fabry disease patients, DRG T2 signal intensity and Lyso-Gb3 level, a marginal, non-significant, positive correlation (Spearman’s *r* = 0.190, *P* = n.s.) was observed.

### DRG volume enlargement is associated with genotype but not with Fabry pain

DRG volume as an imaging biomarker was obtained by voxel-wise MRI DRG morphometry.

**Figure 4:**
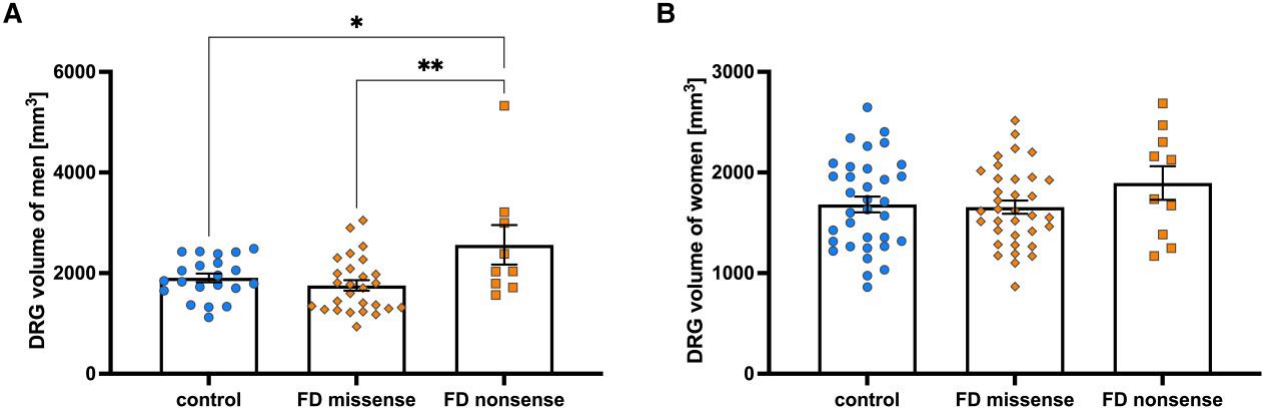
**DRG volume between Fabry disease genotypes**. (**A**) DRG volume is increased in the most severe Fabry disease genotype (nonsense mutation with no residual enzymatic activity) in hemizygous Fabry disease males compared to Fabry disease patients with a missense mutation of GLA and healthy controls (2562 versus 1755 versus 1905 mm^3^). One-way ANOVA: *F* = 5.4; *P* = 0.007; (**B**) but not in heterozygous Fabry disease females (1896 versus 1656 versus 1683 mm^3^). One-way ANOVA: *F* = 1.2; *P* = n.s. DRG, dorsal root ganglion; FD, Fabry disease.

**Figure 5:**
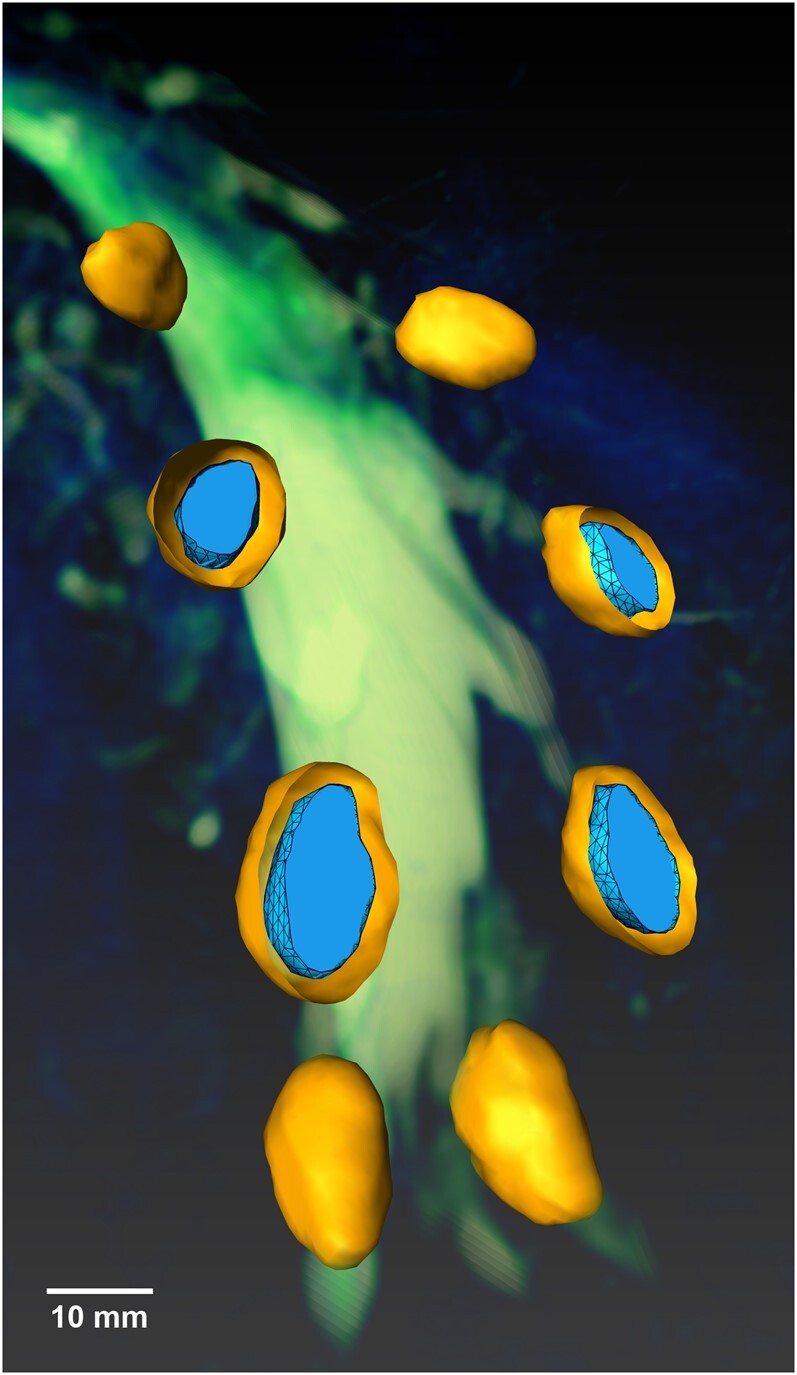
**Visualization of DRG enlargement in Fabry disease**. 3D surface volume-rendered visualization where the L5 and S1 DRGs are intentionally cut to visualize the pathological DRG volume enlargement: The outer shell represents the ground truth voxel-wise segmentation of a representative Fabry disease male with a nonsense mutation. On average, the DRG volumes of this most severely affected genotype show a mean DRG enlargement of +34.5% (outer shell) compared to the mean DRG volume of healthy controls (nucleus). The scale bar applies to the left DRG S1 (DRG bottom left with nucleus). DRG, dorsal root ganglion; L5, lumbar level 5; S1, sacral level 1.

One-way ANOVA showed a difference between Fabry disease males carrying a nonsense mutation, a missense mutation and healthy males (*F* = 5.4; *P* = 0.007). *Post hoc* comparison showed larger DRG volume in Fabry disease males carrying a nonsense mutation compared to Fabry disease males carrying a missense mutation (+46%; 2562 versus 1755 mm^3^; *P* = 0.005; Fig. 4). DRG volume was larger in Fabry disease males carrying a nonsense mutation compared to healthy males (+34.5%; 2562 versus 1905 mm^3^; *P* = 0.034; Figs 4 and 5).

One-way ANOVA showed no difference between Fabry disease females carrying a nonsense mutation, a missense mutation and healthy females (*F* = 1.2; *P* = n.s.). *Post hoc* comparison showed no difference in DRG volume in Fabry disease females carrying a nonsense mutation compared to Fabry disease females carrying a missense mutation (+14.5%; 1896 versus 1656 mm^3^; *P* = n.s.; Fig. 4). DRG volume was not different in Fabry disease females carrying a nonsense mutation compared to healthy females (+12.7%; 1896 versus 1683 mm^3^; *P* = n.s.; Fig. 4).

Also, DRG volume was not different between the group average for all Fabry disease groups and healthy controls (+4.2%; 1843 versus 1768 mm^3^; *P* = n.s.; *t* = 0.73), as well as for the subgroup of males (+5.7; 2013 versus 1905 mm^3^; *P* = n.s.; *t* = 0.52) and females, respectively (+1.7%; 1711 versus 1683 mm^3^; *P* = n.s.; *t* = 0.29).

One-way ANOVA showed no difference between Fabry disease patients with Fabry pain, without pain and healthy controls (*F* = 2.5; *P* = n.s.). *Post hoc* comparison showed no difference in DRG volume in Fabry disease patients with Fabry pain compared to Fabry disease patients without Fabry pain (+15.8%; 1976 versus 1707 mm^3^; *P* = n.s.). DRG volume was not different in Fabry diease patients with Fabry pain compared to healthy controls (+11.8%; 1976 versus 1768 mm^3^; *P* = n.s.).

Correlation analysis showed no significant correlation between the clinical phenotypes and the DRG volume in Fabry disease patients (*r* = −0.003, *P* = n.s.). The level-specific DRG volumes by subgroup are given in Supplementary Table 2. Figure 5 visualizes DRG volume enlargement on individual level for a representative single Fabry disease patient with a nonsense Fabry disease genotype versus the mean DRG volume of all healthy controls.

## Discussion

Fabry disease is a rare X-linked lysosomal storage disorder characterized by the accumulation of the sphingolipid molecule Gb3. Accumulation occurs predominantly in the DRG.^18^ Fabry disease often manifests in early childhood, with pain being one of the first and most debilitating symptoms. The exact mechanisms underlying Fabry pain are not fully understood, in particular whether direct Gb3 deposition in the primary sensory neurons of the DRG, located within the cell body rich area (CBRA) of the DRG, is the primary cause of pain. Alternatively, indirect mechanisms of DRG injury have been suggested by the experimental observation of pro-inflammatory effects of Gb3 on the DRG and on DRG neurons associated with pain behaviour in animal models.^6,19^ Recent advances in 3D DRG MRI assessment have introduced novel *in vivo* imaging methods.^11,20^ Our group has previously established voxel-wise DRG MRI morphometry for accurate DRG volume quantification.^11^ In this study, we extend this method to measure DRG T2 signal within a precise volume of interest. The DRG T2 signal, which increases with extracellular oedema and intercellular space expansion, is a potential radiologic surrogate for DRG injury.^21^

The objective of this study was to evaluate two imaging end-points, DRG T2 signal and DRG volume, in a large cohort of Fabry disease patients. These end-points are being investigated as potential biomarkers for Fabry disease and, more specifically, for Fabry disease-related pain. It is highly desirable to investigate novel tools and develop measures that facilitate objective assessment of Fabry pain and neuropathic pain syndromes.

Our main finding was the substantial increase in DRG T2 signal in Fabry disease patients with Fabry pain. Compared to healthy controls and Fabry disease patients without Fabry pain, the increase was +39.2% and +29.4%, respectively (Figs 1 and 2). These results suggest an association between increased T2 signal and pain in Fabry disease. Notably, the increase in DRG T2 signal was observed to be consistent among Fabry disease patients with Fabry pain, regardless of sex or genetic variation. The analysis revealed a comparable increase in DRG T2 signal for both nonsense and missense genotypes in patients with Fabry pain, compared to healthy controls. The increases were +38.1% and +39.2%, respectively (Fig. 2).

The relationship between DRG T2 signal and Fabry pain does not appear to be linearly related to absolute Gb3 load. This observation is highlighted by the fact that Gb3 load is markedly higher in genotypes with nonsense mutations, which lack residual enzymatic function of lysosomal alpha-galactosidase. In contrast, missense mutations often retain some degree of enzyme function, yet show a lower Gb3 load. In line with this, we found only a marginal, non-significant, positive correlation between Lyso-Gb3 and DRG T2 signal. In our model predicting Fabry pain across different Fabry disease genotypes, the DRG T2 signal emerged as the only independent predictor besides Lyso-Gb3. The correlation between Lyso-Gb3 and Fabry pain, at least in males, has been shown previously. However, the same study showed that Lyso-Gb3 is not a sufficient biomarker for pain in the context of disease progression.^22^ Our results suggest that DRG T2 signal acts as an independent imaging biomarker for Fabry pain, distinct from Lyso-Gb3 levels. However, the precise pathophysiological mechanisms underlying this association remain to be elucidated.

The most obvious interpretation of the DRG pathology underlying the increased DRG T2 signal as an imaging biomarker of Fabry pain in Fabry disease is DRG extracellular oedema and neuronal loss, both contributing to the expansion of the DRG intercellular space.^21^ This observed increase in DRG T2 signal could be due to several factors. First, it could result from the swelling of sensory neurons, a phenomenon previously observed in the rat knockout model.^23^ Second, the infiltration of immune cells into the DRG, triggering an inflammatory response, as suggested by preclinical studies, could explain the increase in DRG T2 signal.^6^ Schwann cell-induced changes in the excitability of DRG neurons in Fabry disease may also contribute to the increase in T2 signal at the DRG organ level.^24^ In addition, gliosis, similar to that seen in the CNS in Fabry disease, may contribute to increased interstitial water content within the DRG.^25^ This interpretation of microstructural changes in the CBRA is supported by the fact that extracellular oedema and expansion of the intercellular space are typical microstructural features that delay the decay of the MR T2 signal.^21,26^

The hypothesis of direct injury leading to loss of sensory neurons in the CBRA of the DRG in Fabry disease is supported by several experimental findings. The DRG is remarkably well vascularized and its blood–tissue barrier is characteristically permeable. This permeability makes the primary sensory neurons of the DRG particularly vulnerable to toxic metabolites.^27,28^ In a previous study investigating diabetic polyneuropathy, researchers found a correlation between DRG T2 signal and the severity of painful diabetic neuropathy. This study also suggested elevated triglyceride and HbA1c levels as potential contributing factors.^9^ In the CNS of Fabry disease patients, Gb3 has been observed to accumulate predominantly at sites with a compromised blood–tissue barrier.^29,30^ A hallmark of Fabry disease is the significant accumulation of Gb3 within the DRG, where it is found in high concentrations not only intracellularly within the CBRA but also in endothelial, glial and perineural cells.^31,32^

Evidence suggests that alterations in neuronal ion channel expression and function may play a role in sensory impairment and pain in Fabry disease. For example, the *Gla* KO mouse model has shown pathological Gb3 deposition in the CBRA of the DRG. These deposits are associated with increased TRPV1 channel expression in the DRG, leading to increased heat hypersensitivity.^5^

Another study highlighted that Gb3 accumulation not only induces neuronal hyperexcitability in the DRG but also triggers Ca^2+^-dependent excitotoxic neurodegeneration, culminating in altered pain behaviour.^18,33^ These findings underscore the complex interplay between Gb3 accumulation, ion channel dysfunction and sensory pathology in Fabry disease.

An alternative explanation for the effect of Gb3 deposition in Fabry disease is an indirect impairment of DRG sensory neurons, rather than a direct effect. This hypothesis is supported by a human study that found increased cytokine gene expression in peripheral blood mononuclear cells (PBMC) of Fabry disease patients, correlating with the Fabry pain phenotype.^34^ Such pro-inflammatory effects of Gb3 have also been observed.^19^ In addition, in the *Gla* KO mouse model, Gb3 may interact with pattern recognition receptors (PRR) on immune cells. This interaction may lead to a suppressed immune response, characterized by fewer CD206+ macrophages and reduced expression of pro-inflammatory cytokine genes, facilitating clearance of Gb3 from DRG neurons.^6^ This suppressed immune response, possibly a mechanism to prevent exaggerated autoimmune responses, may indirectly influence pain perception. These immune response mediators may be involved in the regulation of DRG sensory neuron ion channel gene expression, thereby contributing to peripheral sensitization and altered pain behaviour.

The accumulation of Gb3 within the microvascular endothelium suggests potential impairment of DRG microvascular function and metabolic regulation. There is evidence that Gb3 deposition significantly affects the endothelial cells of blood vessels serving nerve fibres.^35^ In the CNS, similar endothelial deposition has been associated with microvascular dysfunction and tissue ischaemia.^36^ This phenomenon may also affect the DRG, resulting in microvascular impairment. Notably, studies have shown reduced microvascular perfusion in the DRG of a small cohort of Fabry disease patients.^10^ While the direct relationship between impaired DRG microvascular perfusion and Fabry pain remains a speculative yet intriguing area of study, it underscores the complex role of the DRG in both health and disease.^37^ The DRG’s dense capillary network and extensive blood supply, the functional significance of which is still not fully understood, may be key to unravelling pain mechanisms in Fabry disease. In addition, the DRG may regulate afferent signalling, potentially linking neuronal activity to microvascular perfusion. This regulation may be crucial for maintaining the overall functionality of sensory neurons, including the supply of structural components and neurotransmitters to peripheral nociceptor terminals.^38^

Our second major finding is the confirmation of DRG volume enlargement in Fabry disease. In our large cohort of 89 participants, the observed DRG volume enlargement was less pronounced than previously reported in smaller cohorts.^10,39-41^ Specifically, in the genotype group with the most severe enlargement (nonsense mutations), we observed a +46% increase compared to missense mutations and a +34% increase compared to healthy controls (Figs 4 and 5). This is in contrast to previous reports of up to a +119% enlargement in male Fabry disease patients.^10^ Several factors may contribute to this discrepancy. First, the large sample size of our study, which is rare for this disease, may better capture its heterogeneity. This heterogeneity may also lead to greater variation in DRG enlargement. Methodologically, previous studies estimated DRG volume by geometric approximation based on the diameters of the three main axes.^10,39-41^ In contrast, our study used voxel-wise segmentation to obtain precise DRG volume measurements across ∼8600 DRG imaging slices. Although the same method was applied to both Fabry disease patients and healthy controls in each respective study, thus minimizing methodological effects on relative enlargement comparisons, differences in study group composition with respect to genotype heterogeneity and sex may explain the variation in volume estimates. Our study not only achieved precise voxel-wise DRG volume measurements but also established distinct genotype categories through comprehensive determination of *GLA* gene mutation types.

As a novel finding, we observed that pathological DRG volume enlargement strongly depended on the severity of the Fabry disease genotype. We found that the extent of DRG volume enlargement varied among Fabry disease genotype groups, with the most pronounced increase seen in patients carrying the nonsense mutation genotype. This effect was particularly evident in male patients (hemizygous) due to the X-chromosomal inheritance pattern of Fabry disease, whereas it was not observed in female patients (heterozygous). These findings suggest that DRG volume enlargement is particularly pronounced in patients harbouring nonsense mutations, which are associated with a lack of GLA enzyme activity. This suggests that DRG volume is intrinsically linked to the level of GLA activity and thus to the burden of Gb3 accumulation.

In summary, we have studied one of the largest cohorts of rare patients with Fabry disease, characterizing them in depth both clinically and genotypically, to investigate novel imaging biomarkers derived from DRG MRI. Our study has the following limitations. First, due to the inclusion of several different Fabry disease genotypes, our large cohort is relatively heterogeneous. Second, we examined only the bilateral lumbosacral L5 and S1 DRGs, which are considered to be the two most relevant functional levels of the lower extremities. The contribution of patients to the pain and non-pain groups was based on the FPQ. No additional testing (e.g. quantitative sensory testing or sural nerve biopsy) was performed to quantify peripheral nerve damage or neuropathy. Furthermore, it was beyond the scope of this study to account for heterogeneous Fabry disease-specific treatments (e.g. enzyme replacement) or for treatment duration, and our observation was only cross-sectional. However, other studies have suggested that Gb3 deposition can be detected intrauterine, so starting therapy in young adulthood may not have a relevant impact, at least regarding Gb3 deposition as a primary event.^42^ Finally, DRG T2 signal remains a surrogate marker for Fabry pain in this study. It may help to explore pathophysiological mechanisms on a molecular or functional electrophysiological basis, but cannot directly reveal them. Therefore, future age- and sex-matched studies should further investigate changes in DRG T2 signal and its underlying mechanisms in Fabry disease.

A context is emerging for Fabry pain where the pain phenotype appears to be directly related to MR imaging pathology *in vivo* at the DRG level. Both novel imaging biomarkers at the DRG level (volume and T2 signal) are relevant to improve the pathophysiological understanding of Fabry pain. These emerging observations of DRG MR imaging pathology in Fabry disease but also beyond Fabry disease in neuropathic pain syndromes may open an avenue to better understand the enigmatic role of the DRG in pain disorders.^38^

## Supplementary Material

fcae155_Supplementary_Data

## Data Availability

Raw data were generated at the University Hospital Würzburg. Derived data supporting the findings of this study are available from the corresponding author on request.
