# Supplementary material for: Dorsal root ganglion magnetic resonance imaging biomarker correlations with pain in Fabry disease
Source: Brain Commun. 2024 May 1;6(3):fcae155. doi: 10.1093/braincomms/fcae155 (PMC11095551; doi:10.1093/braincomms/fcae155)
Supplement: fcae155_Supplementary_Data [file fcae155_supplementary_data.pdf]

Supplementary Materials

ID	sex, age at MRI [years]	leucocyte GLA activity [nmol/min/mg] reference: 0.4-1.0	last Lyso-Gb3 level [ng/ml] reference: < 1.0	*GLA* mutation	FD genotype	FD clinical phenotype	FD pain phenotype
1	female, 55	0.34	5.3	c.1025G>T // p.R342L	missense buried	classic	pain
2	female, 58	0.43	5.2	c.1025G>T // p.R342L	missense buried	classic	pain
3	male, 51	0.02	22.1	c. 1021 G>A// p.E341K	missense buried	classic	pain
4	male, 53	0.02	11.6	c.408T>A // p.D136E	missense buried	classic	pain
5	female, 65	0.22	1.7	c.644A>G // p.N215S	missense other	later onset	no pain
6	female, 56	0.30	0.8	c.1196G>C // p.W399S	missense other	benign	pain
7	female, 56	0.41	15.6	c.404C>T // p.A135V	missense buried	classic	no pain
8	female, 45	0.24	5.9	c.408T>A // p.D136E	missense buried	classic	no pain
9	female, 25	0.18	6.1	c.404C>T // p.A135V	missense buried	classic	pain
10	female, 28	0.16	11.3	c.404C>T // p.A135V	missense buried	classic	pain
11	female, 54	0.10	11.7	c.404C>T // p.A135V	missense buried	classic	pain
12	female, 51	0.15	7.6	c.612G>T // p.W204C	missense buried	classic	pain
13	female, 54	0.22	14.1	c.757del // p.I253Lfs*16	nonsense	unknown	no pain
14	male, 31	0.04	45.3	c.757del // p.I253Lfs*16	nonsense	unknown	pain
15	female, 33	0.20	10.9	c.757del // p.I253Lfs*16	nonsense	unknown	pain
16	female, 60	0.38	17.7	c.757del // p.I253Lfs*16	nonsense	unknown	pain
17	male, 22	0.02	72.5	c.994dup // p.R332Kfs*7	nonsense	classic	pain
18	female, 44	0.20	7.0	c.994dup // p.R332Kfs*7	nonsense	classic	pain
19	male, 64	0.32	< LLOQ	c.937G>T // p.D313Y	missense other	benign	no pain
20	female, 34	unknown	0.2	c.937G>T // p.D313Y	missense other	benign	no pain

21	male, 41	0.02	72.0	c.1029_1030 delTC fs*30	nonsense	classic	pain
22	female, 54	0.22	7.5	c.386T>C // p.L129P	missense buried	classic	pain
23	female, 56	0.17	11.8	c.963G>C // p.Q321H, c.964G>A // p.D322N	unclassifiable	classic	no pain
24	male, 42	0.21	1.0	c.427G>A // p.A143T	missense other	benign	no pain
25	male, 51	0.06	17.9	c.927del // p.L310Sfs*7	nonsense	later onset	pain
26	male, 37	0.03	30.3	c.386T>C // p.L129P	missense buried	classic	pain
27	female, 42	0.29	7.8	c.515G>A // p.C172Y	missense active	classic	no pain
28	female, 60	0.14	4.2	c.416A>G // p.N139S	missense other	later onset	no pain
29	female, 58	0.29	6.1	c.416A>G // p.N139S	missense other	later onset	no pain
30	female, 57	0.41	15.0	c.137A>G // p.H46R	missense buried	classic	no pain
31	female, 31	0.15	12.6	c.1069C>T // p.Q357X	nonsense	classic	pain
32	female, 39	0.20	7.5	c.515G>A // p.C172Y	missense active	classic	pain
33	female, 46	0.27	0.7	c.640-16A>G	intron	unknown	no pain
34	female, 57	0.62	unknown	IVS0-10C>T, IVS4-16A>G, IVS6-22 C>T	intron	unknown	pain
35	male, 42	0.04	30.8	c.1000-10G>A	intron	unknown	pain
36	female, 29	unknown	0.8	c.937G>T // p.D313Y	missense other	benign	no pain
37	female, 63	unknown	4.5	c.1000-10G>A	intron	unknown	pain
38	male, 32	0.03	20.0	c.1069C>T // p.Q357X	nonsense	classic	pain
39	female, 45	0.22	12.0	c.568del // p.A190Pfs*2	nonsense	classic	pain
40	male, 21	0.04	38.8	c.568del // p.A190Pfs*2	nonsense	classic	pain
41	female, 48	0.25	10.5	c.363del // p.N122Ifs*8	nonsense	classic	no pain
42	male, 49	0.20	0.7	c.427G>A // p.A143T	missense other	benign	no pain

43	male, 70	0.05	6.7	c.644A>G // p.N215S	missense other	later onset	no pain
44	female, 44	0.22	5.4	c.874G>C // p.A292P	missense buried	classic	pain
45	male, 36	0.17	51.0	c. 155G>C // p.C52S	missense other	classic	pain
46	female, 45	0.41	10.4	c.1250T>C // p.L417P	missense buried	unknown	pain
47	female, 54	0.36	1.9	c.644A>G // p.N215S	missense other	later onset	no pain
48	male, 48	0.20	12.5	c.644A>G // p.N215S	missense other	later onset	no pain
49	female, 48	0.29	0.6	c.937G>T // p.D313Y	missense other	benign	no pain
50	male, 58	0.13	5.4	c.644A>G // p.N215S	missense other	later onset	no pain
51	male, 36	0.03	38.7	IVS2+1 (G>A)	intron	classic	pain
52	male, 56	0.20	4.3	c.644A>G // p.N215S	missense other	later onset	no pain
53	male, 50	0.04	4.6	c.644A>G // p.N215S	missense other	later onset	no pain
54	male, 43	0.04	61.7	c.863delC // p.A288Vfs*29	nonsense	classic	pain
55	female, 78	0.43	1.2	c.427G>A // p.A143T	missense other	benign	pain
56	male, 51	0.02	112.0	c.406G>C // p.D136H	missense buried	classic	pain
57	male, 65	0.24	4.9	c.644A>G // p.N215S	missense other	later onset	no pain
58	male, 60	0.18	3.2	c.644A>G // p.N215S	missense other	later onset	no pain
59	male, 68	0.35	4.9	c.720G>C // p.K240N	missense other	later onset	no pain
60	female, 36	0.30	17.9	c.963G>C // p.Q321H, c.964G>A // p.D322N	unclassifiable	classic	pain
61	female, 70	0.20	2.4	c.644A>G // p.N215S	missense other	later onset	no pain
62	female, 37	0.17	14.1	c.718_719del // p.K240Efs*9	nonsense	unknown	pain
63	female, 59	0.51	1.9	c.644A>G // p.N215S	missense other	later onset	pain
64	male, 33	0.04	31.5	c.973G>A // p.G325S	missense other	later onset	pain

65	female, 21	0.24	0.8	c.644A>G // p.N215S	missense other	later onset	no pain
66	male, 29	0.02	50.9	c.1072_1074del // p.G358delE	unclassifiable	classic	pain
67	male, 64	0.11	5.2	c.644A>G // p.N215S	missense other	later onset	no pain
68	male, 32	0.06	2.7	c.644A>G // p.N215S	missense other	later onset	no pain
69	female, 19	0.29	4.9	c.927del // p.L310Sfs*7	nonsense	later onset	no pain
70	female, 46	0.21	2.2	c.644A>G // p.N215S	missense other	later onset	no pain
71	male, 65	0.45	0.5	c.937G>T // p.D313Y	missense other	benign	no pain
72	male, 67	0.05	5.1	c.265C>T // p.L89F	missense buried	later onset	no pain
73	male, 40	0.19	0.7	c.937G>T // p.D313Y	missense other	benign	no pain
74	female, 42	0.27	14.5	c.119C>T // p.P40L	missense buried	classic	pain
75	female, 23	0.42	6.1	c.201_391_delExon2 // p.A73_L131delX	nonsense	classic	no pain
76	male, 68	0.27	0.7	c.427G>A // p.A143T	missense other	benign	no pain
77	male, 19	0.41	0.6	c.937G>T // p.D313Y	missense other	benign	no pain
78	male, 20	unknown	7.7	c.124A>G // p.M42V	missense buried	classic	pain
79	female, 69	unknown	5.4	c.124A>G // p.M42V	missense buried	classic	pain
80	female, 22	0.45	0.6	c.427G>A // p.A143T	missense other	benign	no pain
81	female, 47	0.29	0.6	c.427G>A // p.A143T	missense other	benign	no pain
82	female, 40	0.55	0.6	c.644A>G // p.N215S	missense other	later onset	no pain
83	female, 54	0.35	3.6	c.973G>A // p.G325S	missense other	later onset	no pain
84	male, 44	0.37	0.6	c.937G>T // p.D313Y	missense other	benign	no pain
85	male, 31	0.20	21.2	c.1244T>G // p.L415R	missense buried	classic	pain
86	female, 66	0.43	3.6	c.973G>A // p.G325S	missense other	later onset	no pain

87	male, 39	0.05	139.0	c.1091_1092 delCT // p.365fs*9	nonsense	classic	pain
88	female, 51	0.45	0.5	c.376A>G // p.S126G	missense other	benign	no pain
89	male, 43	0.04	135.0	c.426C>A // p.C142X	nonsense	classic	pain

Supplementary Table 1: FD patient demographics and clinical data.

FD=Fabry disease; GLA=Alpha-galactosidase A; *GLA*=Alpha-galactosidase A gene; Gb3=Globotriaosycleramide.

	DRG Volume L5 [mm^3]	DRG Volume S1 [mm^3]
All FD patients (n=89)	394 ±15	528±25
Sex		
female (n=50)	370±16	486±18
male (n=39)	424±26	582±51
FD genotype (female)		
nonsense-mutation (n=10)	418±48	530±43
missense-mutation (n=35)	358±16	470±21
FD genotype (male)		
nonsense-mutation (n=9)	545±74	736±133
missense-mutation (n=27)	382±24	495±32
FD pain phenotype		
FD pain (n=45)	430±24	559±44
no FD pain (n=44)	357±16	496±21
All healthy controls (n=55)	384±14	500±19

Sex		
female (n=34)	362±17	479±25
male (n=21)	419±24	534±28

Supplementary Table 2: Descriptive analysis of DRG volumes in subgroups of the FD cohort and healthy controls. DRG volumes of the lumbosacral levels L5 and S1 of the respective subgroups of the FD patient group by sex, FD genotype and FD pain phenotype as well as the healthy controls. DRG=Dorsal root ganglion; FD=Fabry disease; L5=lumbar level 5; S1=sacral level 1.
